# Si nanopatterning by reactive ion etching through an on-chip self-assembled porous anodic alumina mask

**DOI:** 10.1186/1556-276X-8-71

**Published:** 2013-02-12

**Authors:** Violetta Gianneta, Antonis Olziersky, Androula G Nassiopoulou

**Affiliations:** 1 NCSR Demokritos/IMEL, Terma Patriarhou Gregoriou, Aghia Paraskevi, Athens, 15310, Greece

**Keywords:** Porous anodic alumina, Si nanopatterning, Reactive ion etching, Fluorine chemistry

## Abstract

**Abstract:**

We report on Si nanopatterning through an on-chip self-assembled porous anodic alumina (PAA) masking layer using reactive ion etching based on fluorine chemistry. Three different gases/gas mixtures were investigated: pure SF_6_, SF_6_/O_2_, and SF_6_/CHF_3_. For the first time, a systematic investigation of the etch rate and process anisotropy was performed. It was found that in all cases, the etch rate through the PAA mask was 2 to 3 times lower than that on non-masked areas. With SF_6_, the etching process is, as expected, isotropic. By the addition of O_2_, the etch rate does not significantly change, while anisotropy is slightly improved. The lowest etch rate and the best anisotropy were obtained with the SF_6_/CHF_3_ gas mixture. The pattern of the hexagonally arranged pores of the alumina film is, in this case, perfectly transferred to the Si surface. This is possible both on large areas and on restricted pre-defined areas on the Si wafer.

**PACS:**

78.67.Rb, 81.07.-b, *61.46.-w*

## Background

Si nanopatterning finds important applications in nanoelectronics, photonics, and sensors. Advanced techniques as electron beam lithography or focused ion beam milling can be used in this respect; however, they are both expensive and time consuming when large areas have to be patterned. The use of a masking layer either on the whole wafer or locally on pre-defined areas on the Si substrate can provide a good and cost-effective alternative to the above techniques. Porous anodic alumina (PAA) thin films on Si offer important possibilities in this respect. PAA films can be fabricated on the Si wafer by electrochemical oxidation of a thin Al film deposited on the Si surface by physical vapor deposition. The so-formed aluminum oxide (alumina) shows highly ordered vertical pores that can reach the Si substrate and can be used either as a masking layer for Si nanopatterning [1-4] or as a template for nanowire and nanocrystal growth within the pores. PAA films can be also used as the dielectric material in metal-oxide-metal (MIM) capacitors [5-7] and as the charging medium in non-volatile memory devices [8]. PAA films can be formed either on large areas or on pre-selected small areas of the Si wafer. This is very useful in all the above applications. In Si nanopatterning, the Al film is first patterned and is then anodized to form the PAA mask. It is thus possible to pattern both small areas and very large areas on the Si wafer.

Perfectly hexagonal self-ordered PAA films were first reported on Al foils by Masuda and Fukuda in 1995 [9]. Other works then followed, which focused on the variation of the main properties of such ordered PAA films, i.e., the cell size, pore diameter, and pore depth as a function of the anodization conditions (i.e., the acidic solution, the anodization voltage, and the anodization time used [10-12]).

For a perfect masking technology for Si nanopatterning, the development of optimized PAA films with tunable pore size and density on the Si wafer are needed. Perfect PAA layers are easily achieved on an Al foil [13,14]. After their release from the Al substrate, free standing PAA membranes are fabricated. Such membranes were used in the literature for Si nanopatterning [15]. However, the direct formation of the PAA mask on the Si substrate offers more flexibility in the etching process than free standing PAA membranes. The structural difference of PAA films on Si compared with similar films on an Al foil is mainly at the interface with the Si substrate. Anodization of the film on Si proceeds as in the case of the Al foil until the so-called barrier layer of the alumina film reaches the Si surface. At this stage, the barrier layer at each pore bottom is detached from the rigid Si substrate under mechanical stress, forming a thin capping layer over a void at each pore base [16,17]. After the void and capping layer formation, if the electrochemical process is not stopped, it proceeds by oxidizing the Si surface, starting from the pore walls and continuing to fully oxidize the Si surface underneath the PAA film. In most of the applications, the anodization has to be stopped just after full Al consumption. The barrier layer at each pore bottom has to be removed so as to get pores that reach the Si surface.

In this paper, we applied optimized PAA thin films on Si with regular long range pore arrangement and we investigated the pattern transfer to the Si wafer using reactive ion etching (RIE) with three different fluorine gas mixtures: pure SF_6_, SF_6_/O_2_, and SF_6_/CHF_3_.

## Methods

PAA films used in this work were fabricated by anodic oxidation of an Al film, deposited on Si by electron gun evaporation. The electrolyte used was an aqueous solution of oxalic acid, 5 w.t.%, and the process was carried out at 1-2°C and a constant voltage of 40 V.

**Figure 1:**
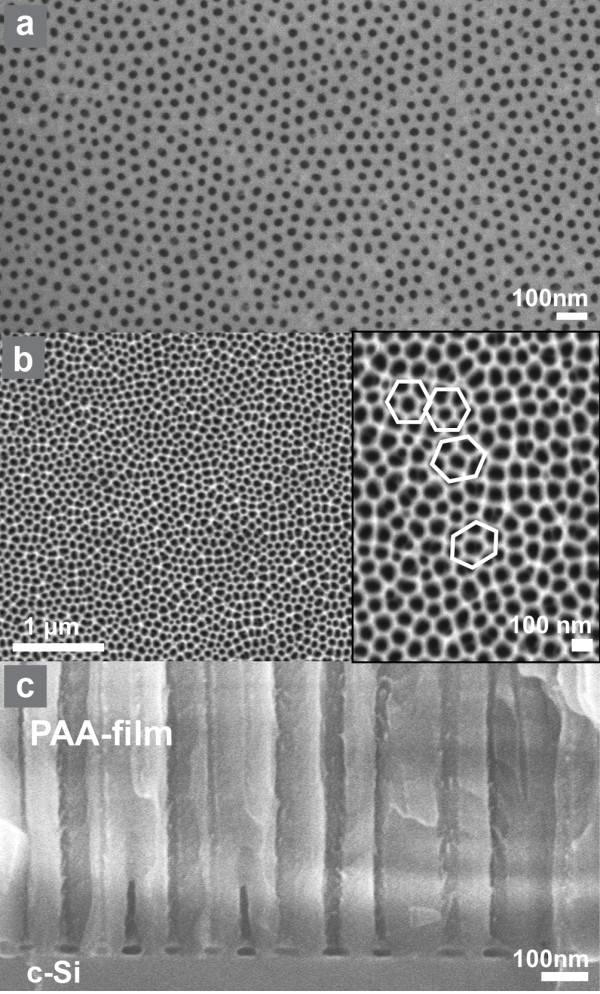
**Examples of SEM images of a PAA film on Si.** The specific PAA film on Si was fabricated by anodic oxidation of an Al film/Si in oxalic acid aqueous solution, using two-step anodization. Images (**a**) and (**b**), and the inset of (**b**) are top view images, while (**c**) depicts a cross-sectional image. The pore diameter in this sample is approximately 40 nm after pore widening for a duration of 40 min.

An example of field emission scanning electron microscopy (SEM) images of a PAA film on Si fabricated by anodizing a 750-nm thick Al film as described above is shown in Figure 1. In Figure 1a, a plane view SEM image of the surface of the as-formed film is depicted, while in Figure 1b, we see a larger area SEM image of the same film after pore widening for 40 min in 0.86 M phosphoric acid. The same film is shown in higher magnification in the inset of Figure 1b, where the hexagonal pore arrangement is clearly depicted and schematically identified in the image.

The pore widening process is performed after the end of the anodic oxidation by immersion of the samples in a 0.86 M phosphoric acid aqueous solution. This process results in partial dissolution of the pore inner wall surface and in the dissolution of the inverted barrier layer at the base of each pore.

In order to improve long range pore ordering of the PAA film, a two-step anodization process was applied in all cases. This process starts with a thick Al film, and part of it is consumed by anodization and alumina dissolution. Pore initiation sites for the second anodization step are thus formed, which help obtain perfect long range pore ordering of the PAA film.

### Pattern transfer to the Si substrate

#### General

Nanopatterning of Si through self-assembled porous anodic aluminum oxide thin films is an interesting lithography-free process for fabricating regular nanoscale patterns on the Si wafer. The area to be patterned can be pre-selected by patterning the Al thin film, which is then anodized using the appropriate conditions. Different processes were reported in the literature for pattern transfer through a PAA film; however, no systematic study was performed to achieve optimized pattern transfer to the Si wafer. Reported works include electrochemical etching of Si through the PAA film [1,3], electrochemical oxidation of Si through the PAA pores, followed by the removal of the PAA film and wet chemical etching of the remaining undulated electrochemical SiO_2_ layer [18,19], and reactive ion etching of Si through the PAA mask using SF_6_ gas or a mixture of CF_4_:Ar:O_2_ gases [20,21]. In most of the above, the patterned features on the Si wafer were very shallow, and the pattern transfer anisotropy was not considered.

In this work, we systematically investigated the etching of Si through a PAA masking layer directly developed on the Si wafer by anodic Al film oxidation. We used reactive ion etching based on fluorine chemistry and, more specifically, we used three different gases: SF_6_, a mixture of SF_6_ and O_2_, and a mixture of SF_6_ and CHF_3_. For these different gases, we examined the etch rate and pattern transfer anisotropy to get all parameters for obtaining the designed pattern.

#### PAA mask formation

The PAA thin films used in this work were formed in oxalic acid aqueous solution (5 w.t.%) at a constant voltage of 40 V. The initial Al thickness was 1.3 μm, deposited by e-gun evaporation. Some of the samples were subjected to an annealing step before anodization (at 500°C for 30 min). In all cases, the anodization was performed in two steps and under the same experimental conditions for all samples. The final PAA thickness was different from one sample to another, depending on the thickness of the sacrificial layer formed during the first anodization step. Three layer thicknesses were used: 390, 400, and 560 nm. The sample characteristics are summarized in Table 1.

**Table 1:** Characteristics of the PAA layers in the three different samples used in this work

	**PAA thickness (nm)**	**Pore size in nm after pore widening for 40 min**	**Annealing**
Sample 1	390	35 – 45	No
Sample 2	560	35 – 55	Yes
Sample 3	400	35 – 45	Yes

**Figure 2:**
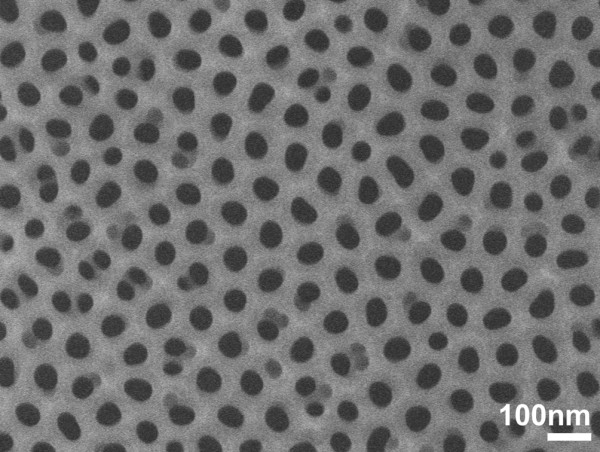
**High magnification top view SEM image of sample 1.** The PAA film thickness of sample 1 is 390 nm, and the average pore diameter is about 45 nm.

All samples were subjected to pore widening and removal of the barrier layer from pore base to get vertical pores that reach the Si substrate. An example of SEM image of the surface of an optimized PAA film used in this work is depicted in Figure 2. In this sample, the Al film was not annealed before anodization. The average pore size was 45 nm, and the PAA film thickness was 390 nm.

#### Reactive ion etching of Si through the PAA mask

The mechanisms involved in reactive ion etching combine physical (sputtering) and chemical etching. The gases or mixture of gases used and the RIE power and gas pressure are critical parameters that determine the etch rate. The etch rate is also different on large Si surface areas compared to the etch rate through a mask with nanometric openings. In this work, the PAA mask used showed hexagonally arranged pores with size in the range of 30 to 50 nm and interpore distance around 30 nm.

Three different gases or gas mixtures were used: SF_6_ (25 sccm), a mixture of SF_6_/O_2_ (25 sccm/2.8 sccm), and a mixture of SF_6_/CHF_3_ (25 sccm/37.5 sccm). In the first case, the etching of Si is known to be isotropic, while in the last two cases, it is more or less anisotropic. Separate experiments were performed for each gas mixture. In all cases, we used three different etching times, namely, 20, 40, and 60 s. The conditions used for the RIE were as follows: power 400 W and gas pressure 10 mTorr.

**Figure 3:**
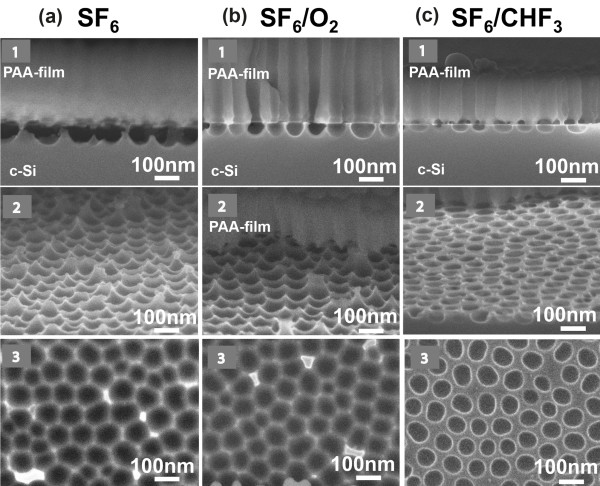
**SEM micrographs of the Si surface of sample 1 after RIE through PAA film and alumina dissolution.** Three different gas mixtures were used for etching, which are indicated on top of the images. The etching duration was 20 s. Micrographs 1 of (**a**), (**b**), and (**c**) are cross-sectional images of the PAA layer/Si stack, while micrographs 2 and 3 are plane view SEM images (slightly tilted in images 2 of (**a**), (**b**), and (**c**); at vertical e-beam incidence in images 3).

An example of SEM image from sample 1 after RIE for 20 s in the three different gases/gas mixtures is shown in Figure 3. Figure 3a corresponds to SF_6_ gas (image 1 is a cross-sectional image of the PAA/Si stack, while images 2 and 3 are plane view images of the nanopatterned Si surface (image 2 at tilted, and 3 at normal incidence) after the removal of the PAA film. Figure 3b,c corresponds to respective images using SF_6_/O_2_ and SF_6_/CHF_3_ gas mixtures. From the images, it is obvious that the etch rate with the SF_6_ gas is higher than the etch rate with the two other gases, and as expected, the etching is isotropic, thus resulting in almost full release of the PAA thin film after RIE of the samples for 20 s. Thus, the nanopatterned Si surface after PAA removal shows quite shallow features. When adding O_2_ to SF_6_, the etching process is slowed down and is more anisotropic. As a result, the 20-s etch time does not lead to PAA mask release, but instead, a nanopatterned surface with features well separated by pore walls are formed (Figure 3b image 1). When replacing O_2_ by CHF_3_, the RIE process is even slower and more anisotropic, thus resulting in shallower etched features at each pore bottom and thicker pore walls, as clearly depicted in Figure 3c.

**Figure 4:**
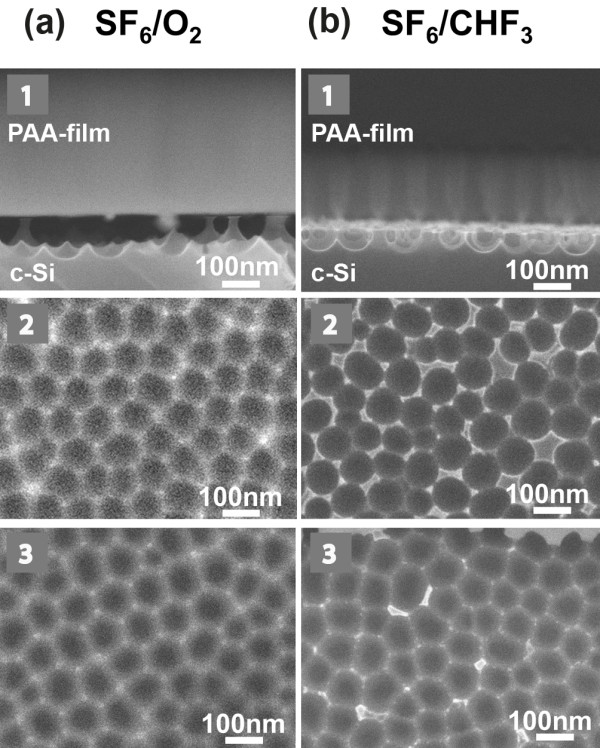
**SEM micrographs of sample 1 after RIE in two different gas mixtures.** Micrographs 1 of (**a**) and (**b**) are cross-sectional images, while images 2 and 3 of (**a**) and (**b**) are plane view images. Etching was performed in SF_6_/O_2_ (images 1 to 3 of (**a**)) and in SF_6_/CHF_3_ (images 1 to 3 of (**b**)). The etching duration was 40 s in images 1 and 2, and 60 s in images 3. In the cross-sectional images, the PAA mask is present; in the plane view images, it is removed, revealing the nanopatterned Si surface.

By increasing the etching time, the depth of the etched holes at each pore bottom increases, but gradually, the lateral etching results in almost full etching of the inter-hole walls, starting from their top, and finally PAA membrane release. This happens earlier with SF_6_ (isotropic etching) than with the other two gas mixtures. Figure 4 depicts the SEM images of sample 1 after etching in SF_6_/O_2_ (Figure 4a) and in SF_6_/CHF_3_ (Figure 4b) for 40 s (images 1 and 2 of Figure 4a, and images 1 and 2 of Figure 4b) and 60 s (images 3 of Figure 4a,b). Already for 40-s etching time, the PAA film is fully released when using SF_6_/O_2_ gas mixture, and the Si inter-hole walls are reduced in height. With SF_6_/CHF_3_ gas mixture, well-separated nanofeatures on Si are formed, with the inter-pore Si walls intact at their top surface. Finally, for the 60-s etching time, the PAA layer was fully released in both gas mixtures.

**Figure 5:**
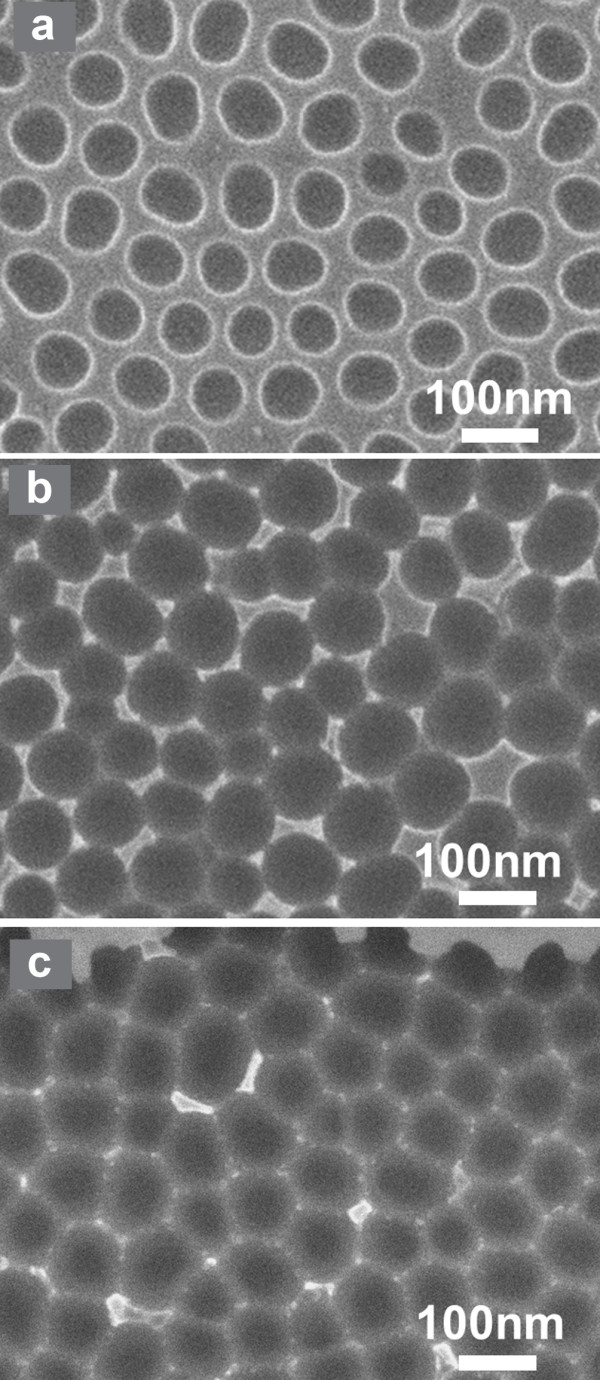
**Plane view SEM images of the Si surface of sample 1.** The images show the nanopatterned Si surface of sample 1 after etching through the PAA mask using SF_6_/CHF_3_ gas mixture for 20 s (**a**), 40 s (**b**), and 60s (**c**). The alumina film was removed before observation.

Etching evolution with time in SF_6_/CHF_3_ is illustrated by the SEM images of the nanopatterned Si surface after the removal of the PAA mask, depicted in Figure 5 for 20-, 40- and 60-s etching time. Due to the reduced etch rate and process anisotropy, pattern formation is more controllable than with the SF_6_ or SF_6_/O_2_.

### Effect of Al annealing before anodization

**Figure 6:**
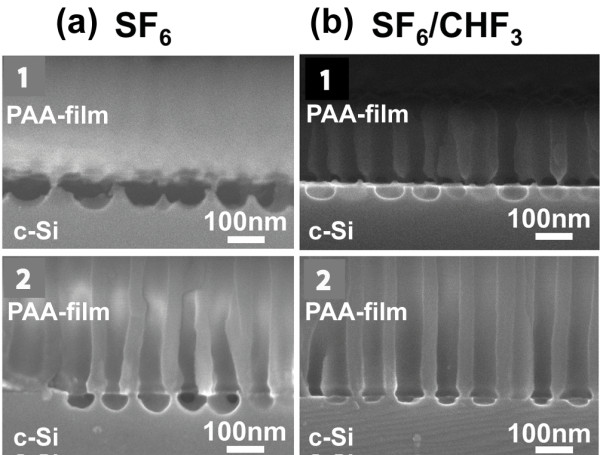
**Cross-sectional SEM images of two samples.** One non-annealed and one that was annealed in nitrogen gas before anodization. Cross-sectional view of sample 1 that was not annealed (images 1 of (**a**) and (**b**)) and sample 2 that was annealed at 500°C for 30 min in nitrogen gas before anodization for alumina formation (annealed; images 2 of (**a**) and (**b**)). Etching was performed for 20 s in SF_6_ (images 1 and 2 in (**a**)) and SF_6_/CHF_3_ (images 1 and 2 in (**b**)), respectively.

**Figure 7:**
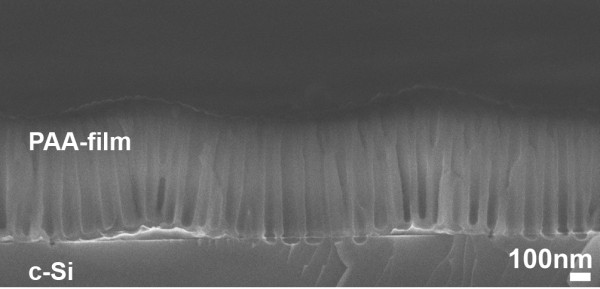
**Alumina/Si interface of the Al-annealed sample.** Cross-sectional SEM image of the interface PAA/Si of an Al-annealed sample at 500°C for 30 min in nitrogen gas. An undulation of the interface is depicted, attributed to Al diffusion into Si (due to the annealing) before anodization.

Good adhesion of the Al film with Si is important for obtaining a sharp interface between the PAA film and Si. The adhesion of the PAA film with Si is an important parameter for achieving etching anisotropy. If adhesion is not good, the reactive gases enter underneath the PAA mask through the alumina pores and start to etch the whole Si surface, resulting in mask release. In order to avoid this effect, an annealing step of the Al film at 500°C for 30 min before electrochemical oxidation was used in samples 2 and 3. The effect of Al annealing is illustrated in Figure 6 by comparing sample 1 (non-annealed; images 1 of (a) and (b)) with sample 2 (annealed; Figure 6, images 2 of (a) and (b)) after etching for 20 s in SF_6_ (Figure 6, images 1 and 2 of (a)) and SF_6_/CHF_3_ (Figure 6, images 1 and 2 of (b)), respectively. We observe that in the case of the non-annealed sample, there is a full detachment of the PAA mask in SF_6_ gas and partial detachment in SF_6_/CHF_3_. The difference between the two cases is due to the higher etch rate with SF_6_ compared with SF_6_/CHF_3_ and the isotropic nature of the process in the case of the SF_6_ gas. When the Al film is annealed before PAA formation, in both cases of gases, under the same etching conditions as for the non-annealed sample, there is no PAA detachment from the Si substrate. This is attributed to the better adhesion of the Al film to the Si substrate. On the other hand, the annealing created an undulation of the PAA film/Si interface. This is illustrated in the cross-sectional SEM image of the PAA/Si stack of a sample annealed at 500°C before Al electrochemical oxidation (Figure 7). This interface undulation is attributed to the fact that Al annealing results, in general, in Al diffusion into the Si substrate and local creation of spikes. This is a well known phenomenon in microelectronics, which causes junction failure when using Al metallization on shallow junctions. Al diffusion into Si introduces some roughness between the Al film and the Si substrate that can result in an undulation of the PAA layer/Si interface.

## Results and discussion

Under the plasma conditions used, the etch rate in SF_6_ gas measured on large patterned areas (100 × 100 μm^2^) is approximately 700 nm/min and etching is isotropic. In the case of etching through the PAA mask, the etch rate was found to be much lower (in the range of 140 to 180 nm/min). This etch rate reduction is expected and is due to the small diameter of the alumina pores (this effect is known as ‘etch lag’).

The addition of O_2_ in SF_6_ is known to result in higher etching anisotropy than with the SF_6_ gas. This is attributed to a different composition of the fluorine-rich polymer formed on the etched Si sidewalls in the case of SF_6_ compared to SF_6_/O_2_, which provides better surface passivation of the etched sidewalls. More specifically, a SiO_*x*_F_*y*_ layer is formed at the etched Si sidewalls when SF_6_ is used. By adding O_2_ to the SF_6_ gas, the number of fluorine atoms in the above fluoropolymer decreases and the number of oxygen atoms per Si increases, thus leading to a more resistant passivation layer on the etched sidewalls and a better etching anisotropy. In the case of our experiments, better anisotropy was observed with SF_6_/O_2_ compared with SF_6_; however, the etch rate in both cases was quite similar. This is illustrated in Table 2 which shows the etch rate with the three different gases in the case of a large area pattern (100 × 100 μm^2^) with a resist mask, compared with the PAA mask pattern.

**Table 2:** **Etch rate of Si through an Al mask compared to a SiO**_**2 **_**mask with large openings**

	**Large area Si etch rate (nm/min)**	**Etch rate through the PAA mask(pore diameter in the range of 35 to 45 nm) nm/min**
SF_6_	700	140 – 180
SF_6_/O_2_	177	140 – 180
SF_6_/CHF_3_	170	65 – 85

Etch rate of Si through a large area (100 × 100 μm^2^) SiO_2_ mask and a 400-nm thick PAA mask with pore diameter in the range of 35 to 45 nm. The difference in the etch rate is attributed to the small size of the etching windows, which is equal to the pore diameter in the case of the alumina mask.

With SF_6_, the etch rate is drastically reduced through the PAA mask compared with the large area etch rate. However, the addition of oxygen in SF_6_ does not create any significant difference in the etch rate compared with SF_6_, as in the case of large area etching. The only effect is a slightly better anisotropy. The significant difference is between these two gases and SF_6_/CHF_3_. In this last case, the etch rate is lower, and better anisotropy is achieved compared to the first two cases.

In general, the mixture SF_6_/CHF_3_ gives highly anisotropic Si etching. This is due to the fact that with the addition of CHF_3_ to the SF_6_ gas, CF_2_ radicals are produced that form a C_*x*_F_*y*_ blocking layer on the Si sidewalls during etching [22]. This thin fluorocarbon polymer limits the rate at which fluorine radicals from the plasma reach the Si surface. In addition, it limits the rate of diffusion of volatile SiF_*y*_ species into Si and, therefore, slows down the chemical etching. Concerning the etch rate in SF_6_/CHF_3_, it is lower compared with both SF_6_ and SF_6_/O_2_ gases. This is due to the fact that the F-atom density is barely higher in this mixture compared to the two other cases, thus retarding Si etching [23].

In Table 2, a comparison is made between the etch rate of a 100 × 100 μm^2^ Si area formed using a resist mask and the etch rate of Si through the PAA mask (pore diameter in the range of 35 to 45 nm). The thickness of the PAA mask was 400 nm. Several samples were considered, and the range of given values is an average of all measured values. As described above, the etch rate is similar with SF_6_ and SF_6_/O_2_, while it is lower with SF_6_/CHF_3_. By increasing the PAA mask thickness from 400 to 500 nm, the etch rate in SF_6_/CHF_3_ was reduced from approximately 70 to 50 nm/min.

Table 3 shows the feature etch depth on nanopatterned Si surface for the three different PAA layer thicknesses and the three different etching times. The first PAA layer was 390-nm thick, and no Al annealing was used before PAA formation. The two other layers were 400- and 560-nm thick, respectively, and an annealing step at 500°C for 30 min was applied to the Al film before anodization. We have observed that although the annealing resulted in a better adhesion of the PAA layer on the Si surface (no detachment even after 60 s of etch time), it also created an undulation of the PAA/Si interface, which led to etching inhomogeneities on the Si surface. In these two last cases, the etch depth varied from zero (non-etched areas) to the maximum value indicated in Table 3. In the case of the non-annealed sample, the etch depth was homogeneous in the whole film. The problem was that for an etching time above 40 s, the lateral etching of the Si film underneath the mask led to mask detachment. The maximum etch depth achieved in that case was around 45 nm.

**Table 3:** **Feature etch depth using SF**_**6**_**/CHF**_**3**_

**PAA layer thickness (nm)**	**Etching time (s)**		
	**20**	**40**	**60**
390 (non-annealed)	32 nm	45 nm	20 nm (lower due to partially etched walls)
400 (annealed)	28 nm	45 nm	56 nm
	(maximum)	(maximum)	(maximum)
560 (annealed)	16 nm	23 nm	45 nm
	(maximum)	(maximum)	(maximum)

Feature etch depth on nanopatterned Si surface through a PAA layer for three different PAA layer thicknesses and three different etching times. The first PAA layer was 390-nm thick, and no Al annealing was used before PAA formation. The two other layers were 400- and 560-nm thick, respectively, and an annealing step at 500°C for 30 min was applied to the Al film before anodization.

## Conclusions

We investigated in detail the RIE of Si through a PAA mask for surface nanopatterning using SF_6_, SF_6_/O_2_, and SF_6_/CHF_3_ gases/gas mixtures. It was found that in all cases, the etch rate through the PAA mask was significantly lower than that on non-masked areas. The smallest etch rate and best anisotropic profiles were obtained with the SF_6_/CHF_3_ gas mixture. Using a PAA mask with highly ordered hexagonally arranged nanopores, a perfect pattern transfer of the nanopores to a large Si area is achieved. The same is possible on small pre-defined areas on the Si wafer.

## Competing interest

The authors declare that they have no competing interests.

## Authors’ contributions

VG performed the experiments of alumina formation and designed the clean room processes that were performed by the clean room operators. AO obtained the SEM images, and AGN supervised the work, drafted and edited the paper. All authors read and approved the final manuscript.

## Authors’ information

VG and AO are post-doctoral researchers. AGN is the director of research at NCSR Demokritos/IMEL and the head of the “Nanostructures for Nanoelectronics, Photonics and Sensors” research group.
